# Sevoflurane postconditioning is not mediated by ferritin accumulation and cannot be rescued by simvastatin in isolated streptozotocin-induced diabetic rat hearts

**DOI:** 10.1371/journal.pone.0211238

**Published:** 2019-01-25

**Authors:** Hilbert Grievink, Natalia Kuzmina, Mordechai Chevion, Benjamin Drenger

**Affiliations:** 1 Department of Anesthesiology and Critical Care and Pain Medicine, Hadassah Hebrew University Hospital, Jerusalem, Israel; 2 Department of Biochemistry and Molecular Biology Hebrew University of Jerusalem, Jerusalem, Israel; 3 Cyclotron/Radiochemistry/MicroPET Unit, Hadassah Hebrew University Hospital, Hadassah Medical Organization, Jerusalem, Israel; Indiana University School of Medicine, UNITED STATES

## Abstract

Sevoflurane postconditioning (sevo postC) is an attractive and amenable approach that can protect the myocardium against ischemia/reperfusion (I/R)-injury. Unlike ischemic preconditioning (IPC), sevo postC does not require additional induced ischemic periods to a heart that is already at risk. IPC was previously shown to generate myocardial protection against I/R-injury through regulation of iron homeostasis and *de novo* ferritin synthesis, a process found to be impaired in the diabetic state. The current study investigated whether alterations in iron homeostasis and ferritin mRNA and protein accumulation are also involved in the cardioprotective effects generated by sevo postC. It was also investigated whether the protective effects of sevo postC in the diabetic state can be salvaged by simvastatin, through inducing nitric oxide (NO) bioavailability/activity, in isolated streptozotocin (STZ)-induced diabetic hearts (DH). Isolated rat hearts from healthy Controls and diabetic animals were retrogradely perfused using the Langendorff configuration and subjected to prolonged ischemia and reperfusion, with and without (2.4 and 3.6%) sevo postC and/or pre-treatment with simvastatin (0.5 mg/kg). Sevo postC significantly reduced infarct size and improved myocardial function in healthy Controls but not in isolated DH. The sevo postC mediated myocardial protection against I/R-injury was not associated with *de novo* ferrtin synthesis. Furthermore, simvastatin aggravated myocardial injury after sevo postC in STZ-induced DHs, likely due to increasing NO levels. Despite the known mechanistic overlaps between PC and postC stimuli, distinct differences underlie the cardioprotective interventions against myocardial I/R-injury and are impaired in the DH. Sevo postC mediated cardioprotection, unlike IPC, does not involve *de novo* ferritin accumulation and cannot be rescued by simvastatin in STZ-induced DHs.

## Introduction

Myocardial ischemia reperfusion (I/R)-injury is a leading cause of perioperative morbidity and mortality. Protective interventions against I/R-injury include, ischemic-preconditioning (IPC) [1] and -postconditioning [2]. Pharmacological agents such as sevoflurane (sevo) can also trigger cardioprotection [3–7]. Distinct mechanistic overlaps can be achieved by pharmacological agents, such as sevo [8].

Protection of the heart is of supreme importance in disease states which aggravate ischemic heart disease, such as diabetes mellitus (DM). However, the diabetic state interferes with the intrinsic adaptive and cardioprotective mechanisms, such as myocardial preconditioning (PC) and postconditioning (postC), thus enabling cell injury and apoptosis [5, 9, 10]. Interestingly, studies have shown that the diabetic heart (DH) is still amenable to protection, by for example isoflurane PC, but has an increased threshold for activation of its protective mechanisms [11]. Several mechanisms have been found to be involved in the observed resistance for cardioprotection in the DH [12–14]. These include the dysregulation of the mitochondrial permeability transition pores (mPTP), down regulation of prosurvival pathways (phosphoinositide 3-kinase (PI3K)/AKT, extracellular signal related kinase (ERK), with their subsequent effects on mitochondrial adenosine triphosphate–dependent potassium (mK_ATP_) channels [5, 13, 15, 16], and increased receptor activities for pharmacological agents (associated with impaired Janus kinas 2 (JAK2)/AKT signalling) [17].

Many of the adverse consequences of DM and hyperglycemia, and the impaired cardioprotective mechanisms, are thought to result from the combination of reduced nitric oxide (NO) bioavailability/activity, impaired iron homeostasis and the increased generation of reactive oxygen-derived species (ROS) [9, 10, 18–20]. In line with these observations, experimental studies in diabetic db/db mice demonstrated that the HMG-CoA reductase inhibitor simvastatin can attenuate myocardial I/R-injury, without reducing cholesterol levels, through increasing NO synthase (NOS) enzyme activity and bioavailability [21]. Additionally, simvastatin was found to restore the cardioprotective effects of IPC in hyperglycemic dogs by NO-mediated signaling [22].

Previously our group showed that in the healthy heart, cardioprotection by IPC involves the generation of an iron signal through activation of the proteasome, which results in the accumulation of ferritin (L- & H-ferritin mRNA and protein levels), chelation of harmful redox active iron, and a consequential decrease in ROS-induced oxidative damage [4, 23–27]. NO was found to play a role in myocardial protection and ferritin protein accumulation in the healthy heart, however, the biological effects of NO strongly depends on its concentration and the subsequent identity of its bio-active redox forms [28]. In streptozotocin (STZ)-induced DHs, impaired iron homeostasis was found to lead to loss of the IPC-generated myocardial protection [10].

The current study investigated whether alterations in iron homeostasis and ferritin accumulation are involved in the cardioprotective effects generated by sevo postC, and whether these effects can be salvaged by simvastatin, in the STZ-induced DH, through increasing NO bioavailability/activity.

## Materials and methods

### General

All the experimental protocols were approved by the ‘Institutional Animal Care and Use Committee’ of the Hebrew University of Jerusalem School of Medicine, conforming to the Guide for the Care and Use of Laboratory Animals published by the U.S. National Institutes of Health (NIH Publication No. 85–23, revised 1996). In accordance with the Ethical Approval, the number of animal experiments was minimized where possible.

### Animal preparation

Sprague-Dawley male rats (250–275 g) were housed under standard conditions (12h light/12h dark) and had free access to food (Teklad) and water. The animals were acclimated to the local animal facility for at least four days prior to use in experiments. Diabetes was induced by a single i.p. injection of streptozotocin (STZ; 50 mg/kg body weight, in saline; Sigma Aldrich, St Louis, MO, USA). The Control group was injected intraperitoneally (i.p.) with an equal volume of saline. Typically, three days after STZ injection, glucose level exceeded 300 mg/dL. Animals that maintained high blood glucose in repeated blood test (twice a week) were considered diabetic. In order to have diabetic-associated systemic effects and to allow for diabetic complications to develop, the heart perfusion experiments were conducted 4 weeks after STZ injection. Simvastatin (0.5 mg/kg, i.p.) was injected 48h and 24h (day -2 and -1) before the day of the experiments, to randomly assigned animals [21]. We are aware to the drawbacks of the STZ-model, as it is representing a mixed type I and type II diabetes. However, we considered against using high-fat diet with low dose STZ, because this diet per-se is oppressing postC responses [29, 30]. Furthermore, the employment of the STZ-model allowed for comparisons with previous studies by our group [5, 10].

### Heart perfusion and monitoring of hemodynamic parameters

Rats were injected i.p. with sodium heparin (500 units) and 20 min later with sodium pentobarbital (60 mg/kg). Confirmation of deep anesthesia and an unresponsiveness to pain stimuli was confirmed by a negative paw withdraw reflex. Hearts were then rapidly removed and placed in heparinized ice-cold saline. Subsequently, each heart was cannulated via the aorta and retrogradely perfused at a constant perfusion pressure of an 85 cm water column. The standard perfusate consisted of a modified Krebs-Henseleit (KH) buffer containing (mM) NaCl, 118; KCl, 5.8; CaCl_2_, 2.5; MgSO_4_, 1.2; NaHCO_3_, 25 and glucose, 11.1 [26]. The perfusion buffer was gassed with 95% O_2_ and 5% CO_2_. pH was maintained at 7.4. Hearts were kept in a thermostated glass, at a constant temperature of 37.5°C ± 0.1. In order to assess left ventricular function, a small latex balloon-tipped catheter was inserted into the left ventricle through an incision on the left atrium. Hemodynamic parameters, heart rate (HR), end-diastolic pressure (EDP), developed pressure (DP) and its derivatives (+dp/dt and–dt/dp), were recorded. The work index (WI) was calculated according to WI = DP x HR. All data was processed using a customized version of Labview 7.1 software (National Instruments, Austin TX, USA). Sevo was bubbled at a rate of 1.5 Liter/min into the perfusion buffer using a sevoflurane vaporizer (Sevotec 5; Datex-Ohmeda, Tewksbury, MA, USA). The concentration of the administered sevo was measured using a volatile anesthetic gas monitor (Datex Capnomac Ultima, Division of Instrumentation Corp., Helsinki, Finland). The sevo concentration in the KH-buffer was also verified by gas chromatography (Agilent 7200 GC/Q-TOF MS). Any heart that developed persistent ventricular arrhythmias before the ischemic period was excluded from the study.

### Experimental protocols

**Fig 1:**
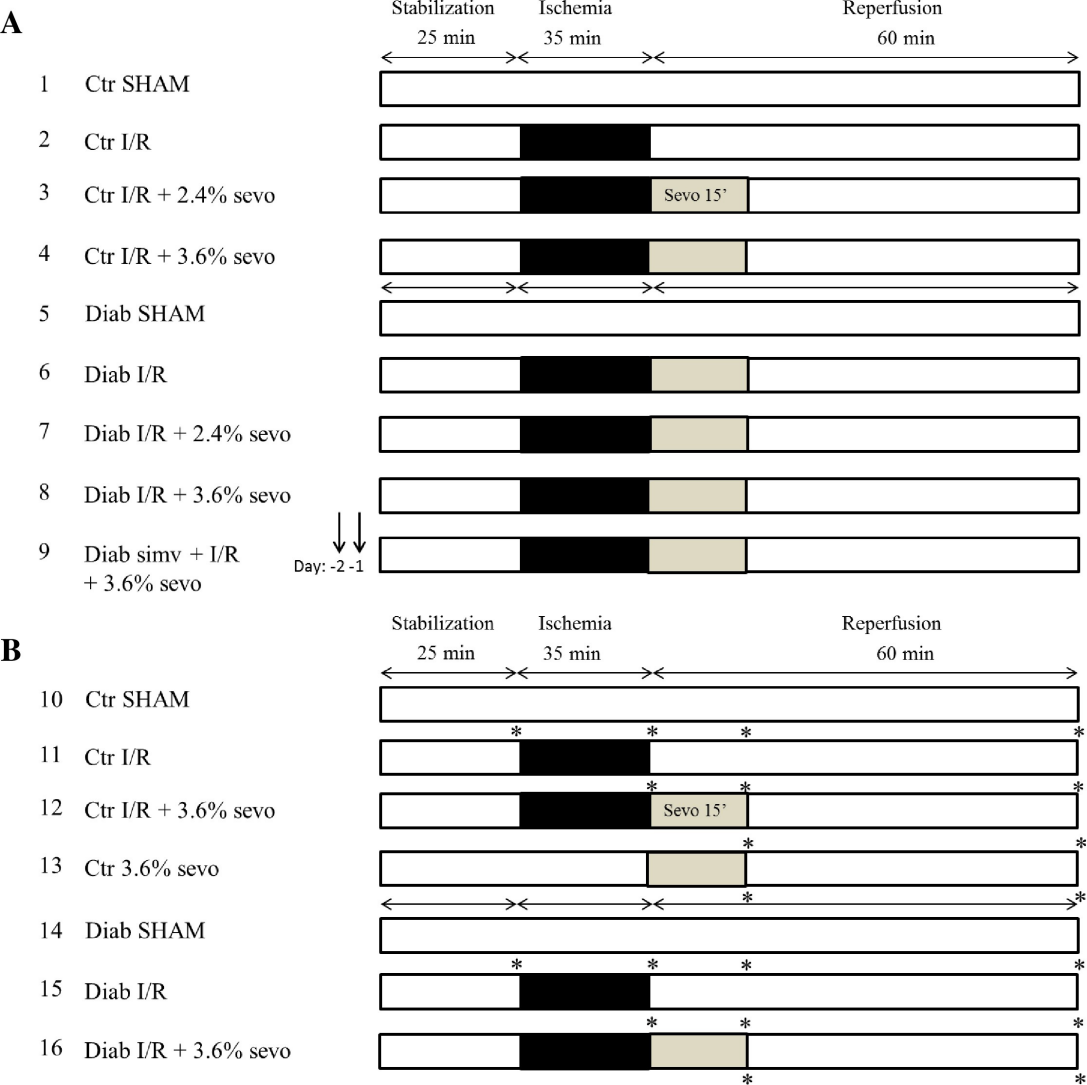
Experimental protocols. (A) Hearts in groups 1–9 were harvested after 120 min (60 min of reperfusion) and stained to allow infarct size determination. Simvastatin (simv) was injected 48h (day-2) and 24h (day-1) before the start of the experiments. (B) Hearts in groups 10–16 were harvested at different time points along the perfusion protocol (*) to allow molecular biological analyses. Ctr = hearts from normoglycemic animals, Diab = hearts from STZ-induced diabetic animals, SHAM = continues perfused normoxia treated hearts, I/R = ischemia/reperfusion, sevo = sefoflurane.

After a stabilization period of 25 min, rat hearts were randomly assigned to one of the experimental groups, as shown in Fig 1. Hearts that were subjected to I/R (n = 6–9 per time point, except simvastatin treated animals; n = 4) were exposed to 35 min of global ischemia followed by 60 min of reperfusion. Normoxic hearts (SHAM and sevo, n = 4 per time point) were continuously perfused with KH solution for an additional 95 min. Sevoflurane (2.4% and 3.6%) was given to the respective groups, for a duration of 15 min, according to Fig 1. Hearts used for infarct size analyses were perfused for 120 min (Fig 1A). Hearts used for molecular biological analyses (ferritin protein levels, eNOS, iNOS, and L- and H-ferritin mRNA expression levels) were harvested and snap frozen in liquid nitrogen at four different time points along the experiments (Fig 1B): (i) end of stabilization (25 min; n = 4), (ii) end of ischemia (60 min; n = 6), (iii) 15 min of reperfusion with or without sevo (75 min; n = 6) and (iv) at the end of reperfusion (120 min; n = 6).

### Infarct size analyses

At the end of reperfusion, hearts were frozen for 15 min at -20°C before slicing into five transverse slices, parallel to the atrioventricular groove. After removing right ventricular and atrial tissue, heart slices were incubated for 30 min in a 1% solution of triphenyltetrazolium chloride at 37°C to differentiate infarcted (pale) from viable (bright red) myocardial area [10]. The size of the infracted tissue was digitally photographed with a Nikon Coolpix 5000 camera and quantified with IMAGE J 1.32 (NIH, USA) software. Determination of the area of infarction was performed by a blinded investigator.

### RNA isolation and cDNA synthesis

Total RNA of was isolated from 100 mg of left ventricular heart tissue using TriReagent (MRC, Cincinnati, OH, USA) according to the manufacturer’s protocol. Isolated RNA was quantified using UV spectrophotometry (Nanodrop, Thermo Scientific, USA) and RNA degradation was precluded by gel electrophoresis. 1 μg of total RNA was reverse transcribed to cDNA using the M-MLV reverse transcriptase (Promega, Madison WI, USA).

### Real-time PCR

Changes in mRNA expression levels were measured using FastStart Universal SYBR Green Master (ROX) (Roche, Mannheim, Germany) and the AB7500 Real-Time PCR System (Applied Biosystems) according to the 2(-ΔΔCT) method [31]. Primers for the mRNA target genes (eNOS, iNOS, H-ferritin and L-ferritin) and the housekeeping gene (β-actin) were designed using primer3 software (http://biotools.umassmed.edu/bioapps/primer3_www.cgi) and are listed in Table 1.

**Table 1:** Primers used for real-time PCR.

Gene	Accession No.	Forward primer	Reverse primer	Exon
β-Actin	NM_031144	TTCCTTCCTGGGTATGGAATC	CGGATGTCAACGTCACACTT	3–4:4
eNOS	NM_021838.2	AAGTGGAAGCTGAGGTGGTG	TGCAGTCCCGAGCATCAAAT	4–5
iNOS	NM_012611.3	GAAGAGACGCACAGGCAGAG	CACACGCAATGATGGGAACT	21–22
Ft-H	NM_012848	ACGTCTATCTGTCCATGGTCTTG	AAAGTTCTTCAGGGCCACAT	1–2:2
Ft-L	NM_022500	CCTACCTCTCTCTGGGCTTCT	CTTCTCCTCGGCCAATTC	1–2:2

### Preparation of heart tissue extracts

Lysis buffer containing 1% deionized Triton X-100 and 0.1% sodium azide in 50 mM Tris-HCl pH 7.5 was incubated with Chelex-100, for a minimum of 24h. Phenyl-methyl-sulfonyl-fluoride (0.25M) and a protease inhibitor cocktail (Roche, Mannheim, Germany) were added immediately prior to use. 100 mg of left ventricular heart tissue was crushed in liquid nitrogen and homogenized in 1 mL of lysis buffer using a Cole Parmer Teflon homogenizer. Subsequently, tissue homogenates were sonicated for 1 min. and incubated on ice for 30 min, while vortexing every 5–10 min. Samples were then centrifuged for 5 min. at 10,000 xg and supernatants were analyzed for total protein content using the Lowry method.

### Heart ferritin levels determination by ELISA

Heart ferritin levels in the cytosolic fraction were determined using the previously described ELISA-based method [32]. Briefly, goat anti-rat liver-Ft was diluted in 0.1 M carbonate–bicarbonate buffer pH 9.6 (Coating buffer). 0.2 mL/well was added to a F96 Maxisorp NUNC-IMMUNO Plate (Nunc, Roskilde, Denmark). After incubation (1h at 37°C followed by 4°C overnight), plates were washed four times with Wash buffer (0.02 M phosphate-buffered saline (PBS), containing 0.1% (w/v) BSA, 0.05% (v/v) Tween 20, and 0.01% (w/v) NaN_3_). Subsequent blocking was performed by adding 0.2 mL of Blocking buffer to each well (0.02 M PBS, 0.01% (w/v) NaN3, and 0.5% (w/v) gelatine). Plates were incubated and washed as described above. Next, 0.2 mL of protein samples or standards, diluted in Dilution buffer (0.02 M PBS containing 0.5% (w/v) BSA and 0.05% (w/w) Tween 20), were added to the wells. Plates were incubated and washed as previously described. After that, 0.2 mL/well of rabbit anti-rat heart-Ft in Dilution buffer was added, and plates were incubated for 1h at 37°C, followed by a 2h incubation at 4°C. After washing the plates, 0.2 mL of goat anti-rabbit γ-globulin-antibodies, conjugated to β-galactosidase in Conjugation buffer (0.01 M phosphate buffer pH 7.6, 10 mM NaCl, 0.1% BSA, 4% PEG 6000, 2 mM MgCl2, and 0.1% NaN3) was added. Plates were incubated for 1h at 37°C and washed four times before adding the substrate (0.2 mL/well). The substrate, chlorophenol-red β-d-galactopyranoside (Roche diagnostic GmbH, Manheim, Germany) was diluted in Substrate buffer (0.01 M phosphate buffer, pH 7.2, 10 mM NaCl and 2 mM MgCl_2_) and incubated at 37°C until color development. Absorbances were read in a microplate reader (MR 5000 Dynatech Laboratories, Chantilly, VA, USA). A primary filter with a peak transmission at 570 nm and a secondary filter with a transmission at 620 nm, were used.

### Statistics

Data are presented as Mean ± SEM. Statistical analyses between values of the same group at various stages of the protocol were performed by one-way analyses of variance (ANOVA). Between groups comparisons were made for each time point using a one-way ANOVA followed by the Dunnett’s post-hoc test, where appropriate. Changes were considered statistically significant when p<0.05.

## Results

Average body weights (BW) and heart weights (HW) were not significantly different between the control animals (Mean ± SEM: BW; 309 ± 4 g, HW; 1.33 ± 0.02 g) and the STZ-induced diabetic animals (Mean ± SEM: BW; 307 ± 6 g, HW; 1.33 ±0.03 g), prior to the *ex vivo* perfusion experiments. Blood glucose levels, in the fed state, were significantly higher for STZ-induced diabetic animals (Mean ± SEM: 539 ± 12 mg/dL, p<0.01) compared to control animals (Mean ± SEM: 126 ± 3 mg/dL).

### Infarct size analyses

**Fig 2:**
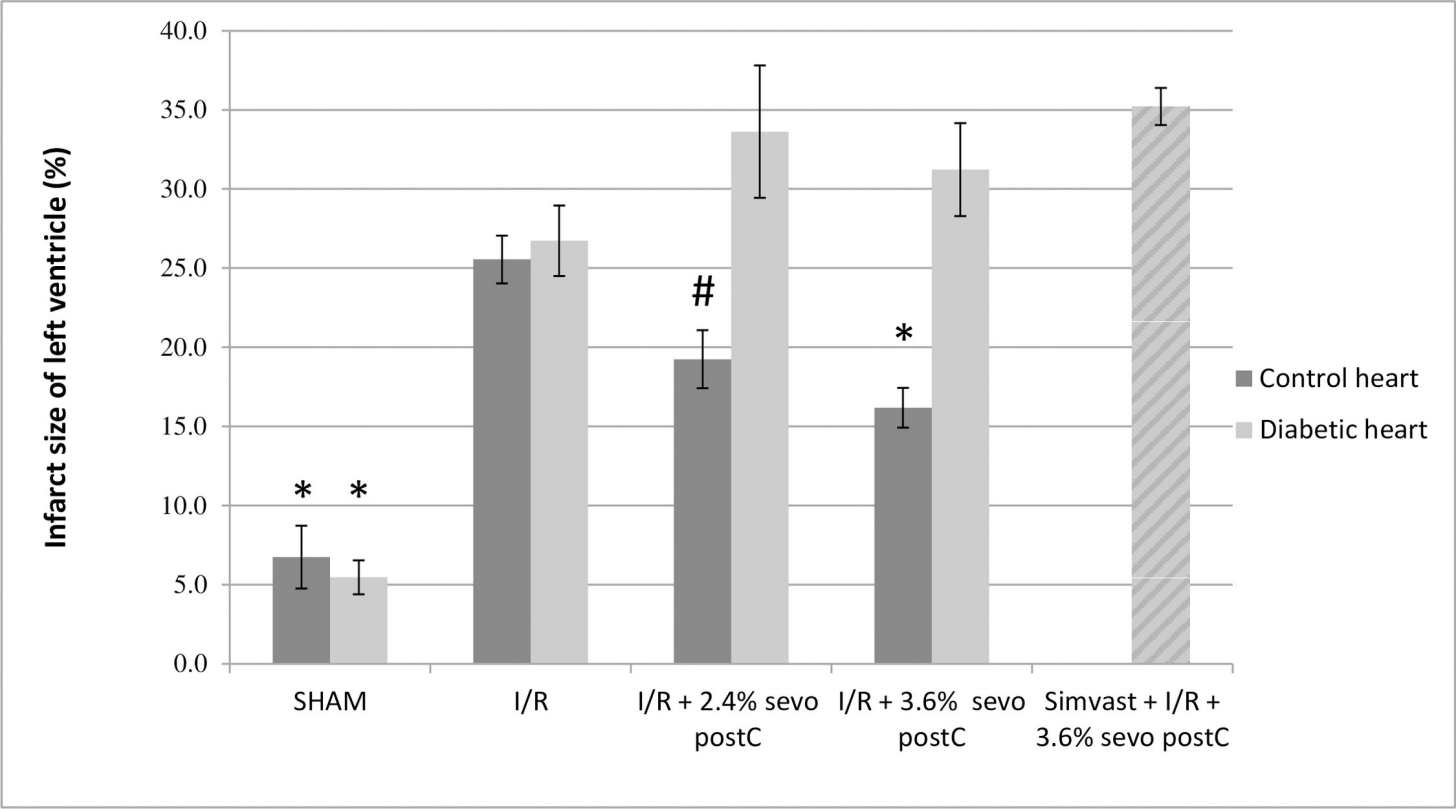
Myocardial infarct size analyses after the different perfusion protocols. Myocardial infarct sizes of the normoglycemic Control hearts are depicted by the dark grey bars. Myocardial infarct sizes of the DHs are depicted by the light grey bars. The DHs with simvastatin and I/R+3.6% sevo postC are depicted by the striated light grey bar. Data are presented as Mean ± SEM, * denotes p<0.01 vs. I/R in the respective group (Control heart or Diabetic heart). # denotes p<0.05 vs. I/R.

As depicted in Fig 2, in normoglycemic Control hearts myocardial infarct sizes were found to be significantly reduced in a dose-dependent manner by sevo postC with 2.4% (19.2% ± 1.8, p<0.05) and 3.6% sevoflurane (16.2% ± 1.3, p<0.01), compared to I/R alone (25.5% ± 1.5). In DHs, 2.4% and 3.6% sevo postC did not reduce myocardial infarct sizes, compared to heart treated by I/R alone (33.6% ± 4.2 and 31.2% ± 2.9, respectively, compared to 26.7% ± 2.2 in I/R group.). As expected, SHAM perfused Control hearts (6.7% ± 2.0, p<0.01) and DHs (5.5% ± 1.1, p<0.01), were found to have significantly lower myocardial infarct sizes, compared to I/R. All further experiments were conducted using 3.6% sevoflurane, as this concentration was found to generate more reduced infarct sizes.

Administration of 0.5 mg/kg simvastatin, to STZ-induced diabetic animals, did not affect the increase in infarct size in DHs subjected to I/R + 3.6% sevo PostC (35.2% ± 1.2), compared to DHs treated by I/R alone (26.7% ± 2.2). The effect of simvastatin on Control hearts was not investigated due to animal care committee restrictions on number of study groups.

### Hemodynamic parameters

Table 2 represents the hemodynamic data and recoveries of the Control hearts and the DHs, after continues perfusion (SHAM), or exposure to I/R with/without 3.6% sevo postC, and/or simvastatin pre-treatment. There were no significant differences in baseline values for HR, EDP, WI and left ventricular DP or its derivatives, +dp/dt and–dp/dt, *within* the groups of Control hearts or DHs. Lower hemodynamic baseline values were found *between* the two groups (Control vs. Diabetic) for several of the recorded parameters. The most prominent difference between the two groups was that DHs have a lower heart rate (mean HR 242.0 bpm ± 3.7, compared to 281.3 ± 6.3 in Control, p<0.01). Depending on the protocol, WI, +dp/dt and -dp/dt baseline values were also found to be significantly lower in DHs compared to Controls (p<0.05). When the averaged baseline values of the various perfusion protocols (in Controls or DHs) were combined and analysed, statistical significance (p<0.05) of reduced function in DHs was reached for HR, +dp/dt, -dp/dt and WI in all protocols, compared to Controls.

**Table 2:** Hemodynamic parameters of Control hearts and STZ-induced Diabetic hearts during the different perfusion protocols.

Parameter	Group	Protocol	Baseline (end of stabilization)	Perfusion			
				75 min (end of sevo PostC)	100 min	120 min (end of reperfusion)	
			Raw data	% Recovery versus baseline			
**HR (bpm)**	**Control**	**SHAM**	271.4 ± 4.4	101.9 ± 3.1 #	102.1 ± 3.1		102.4 ± 3.0
		**I/R**	293.1 ± 5.5	71.3 ± 9.5	98.2 ± 7.2		95.7 ± 6.3
		**I/R + 3.6% sevo postC**	279.4 ± 6.3	59.7 ± 5.1	88.3 ± 6.7		99.3 ± 5.8
	**Diabetic**	**SHAM**	249.6 ± 14.9	97.0 ± 3.6 #	98.0 ± 4.0 *		96.7 ± 5.3
		**I/R**	241.7 ± 9.4 ¥	70.6 ± 3.5	82.0 ± 4.8		80.6 ± 4.8
		**I/R + 3.6% sevo postC**	234.8 ± 10.4 ¥	64.9 ± 7.3	80.5 ± 6.5		84.4 ± 3.7 ¥
		**Simvastatin + I/R + 3.6% sevo postC**	254.5 ± 6.9	51.0 ± 9.9	66.3 ± 9.0		74.3 ± 9.5
**DP** **(mm Hg)**	**Control**	**SHAM**	103.0 ± 6.8	86.3 ± 3.4 *	81.9 ± 4.8 *		76.1 ± 3.3 *
		**I/R**	109.4 ± 6.2	37.6 ± 4.8	32.6 ± 3.2		38.0 ± 3.3
		**I/R + 3.6% sevo postC**	102.8 ± 6.4	45.9 ± 4.8	50.3 ± 5.0 *		46.4 ± 4.2
	**Diabetic**	**SHAM**	98.0 ± 7.8	82.9 ± 5.7 *	79.1 ± 6.3 *		75.3 ± 7.1 *
		**I/R**	110.9 ± 4.9	43.9 ± 5.8	44.7 ± 4.5 ¥		50.0 ± 3.7 ¥
		**I/R + 3.6% sevo postC**	98.8 ± 7.6	48.0 ± 6.6	65.1 ± 2.9 * ¥		57.9 ± 5.0
		**Simvastatin + I/R + 3.6% sevo postC**	112.8 ± 10.1	29.6 ± 6.6	40.6 ± 7.6		41.1 ± 6.2
**+dp/dt** **(mm Hg/s)**	**Control**	**SHAM**	3801.4 ± 378.4	103.9 ± 6.0 *	99.4 ± 4.4 *		92.1 ± 4.8 *
		**I/R**	3596.3 ± 147.2	29.0 ± 2.7	29.1 ± 3.1		32.4 ± 2.3
		**I/R + 3.6% sevo postC**	3300.1 ± 140.1	31.3 ± 3.1	39.3 ± 4.5		37.0 ± 4.1
	**Diabetic**	**SHAM**	3286.4 ± 378.8	94.8 ± 4.5 *	91.8 ± 6.0 *		79.3 ± 8.7 *
		**I/R**	3061.9 ± 171.7 ¥	30.5 ± 3.7	38.3 ± 3.4		43.3 ± 4.3 ¥
		**I/R + 3.6% sevo postC**	2666.7 ± 207.1 ¥	32.8 ± 4.3	49.4 ± 3.6 ¥		46.9 ± 3.3
		**Simvastatin + I/R + 3.6% sevo postC**	3302.0 ± 577.9	25.7 ± 5.6	30.2 ± 4.5		30.0 ± 4.4
**-dp/dt** **(mm Hg/s)**	**Control**	**SHAM**	-2463.9 ± 144.2	88.1 ± 2.1 *	80.2 ± 1.4 *		72.3 ± 2.6 *
		**I/R**	-2617.5 ± 106.1	34.8 ± 4.1	31.6 ± 3.4		34.9 ± 2.7
		**I/R + 3.6% sevo postC**	-2512.9 ± 122.0	35.2 ± 2.8	40.3 ± 3.8		39.0 ± 3.0
	**Diabetic**	**SHAM**	-2291.8 ± 251.3	78.2 ± 5.7 *	71.5 ± 6.0 *		62.6 ± 7.8
		**I/R**	-2220.9 ± 135.9 ¥	35.4 ± 5.5	43.1 ± 4.4 ¥		45.0 ± 4.6
		**I/R + 3.6% sevo postC**	-1902.8 ± 184.7 ¥	43.6 ± 5.7	58.4 ± 5.0 ¥		54.1 ± 4.8 ¥
		**Simvastatin + I/R + 3.6% sevo postC**	-2186.3 ± 325.1	34.8 ± 7.3	39.7 ± 7.7		39.7 ± 7.7
**Work index**	**Control**	**SHAM**	27897.0 ± 1705.4	87.6 ± 3.0 *	83.4 ± 5.3 *		78.0 ± 4.2 *
		**I/R**	31894.3 ± 1693.9	28.9 ± 5.6	32.1 ± 3.9		36.4 ± 3.4
		**I/R + 3.6% sevo postC**	28048.1 ± 1442.1	27.5 ± 4.1	44.6 ± 5.5		45.6 ± 4.5
	**Diabetic**	**SHAM**	24450.6 ± 2545.2	79.7 ± 4.7 *	76.5 ± 5.3 *		72.3 ± 7.4 *
		**I/R**	26772.3 ± 1594.6 ¥	31.9 ± 5.2	37.6 ± 4.6		41.1 ± 4.4
		**I/R + 3.6% sevo postC**	23054.9 ± 2005.4 ¥	30.7 ± 5.4	50.7 ± 4.9 ¥		48.2 ± 4.2
		**Simvastatin + I/R + 3.6% sevo postC**	28641.2 ± 2559.5	15.5 ± 4.4	25.0 ± 2.8		29.5 ± 4.8
** **	** **	** **	**Raw data**				
**EDP** **(mm Hg)**	**Control**	**SHAM**	5.6 ± 1.0	2.4 ±1.4 *	2.6 ± 1.3 *		1.7 ± 1.2*
		**I/R**	4.1 ± 1.2	66.0 ± 6.3	60.7 ± 4.2		49.6 ± 5.5
		**I/R + 3.6% sevo postC**	2.0 ± 1.3	47.1 ± 5.3 #	41.4 ± 4.8 *		36.2 ± 4.9
	**Diabetic**	**SHAM**	3.3 ± 0.5	3.5 ± 1.9*	2.3 ± 2.9 *		2.0 ± 3.7 *
		**I/R**	0.6 ± 1.0	70.7 ± 4.9	57.2 ± 4.4		48.1 ± 4.7
		**I/R + 3.6% sevo postC**	0.9 ± 0.8	52.5 ± 4.7 #	44.6 ± 4.8		40.0 ± 4.2
		**Simvastatin + I/R + 3.6% sevo postC**	1.3 ± 2.0	81.3 ± 3.9	75.3 ± 1.3		70.0 ± 1.8 #

Sevo postC hearts, received 3.6% sevo during the first 15 min. of reperfusion. Data are presented as Mean ± SEM.

* denotes p<0.01 vs. I/R in the respective group. # denotes p<0.05 vs. I/R in the respective group, ¥ p<0.05 vs. the respective protocol in Control.

After treatment by I/R, Control hearts recovered to 36.4% ± 3.4 of the initial WI baseline value, at 120 min, whereas DHs recovered to 41.1% ± 4.4. The EDP values were similar in both Control and DHs. Ischemia significantly increased EDP values after reperfusion. Nevertheless, Control and DHs subjected to I/R + 3.6% sevo postC recovered better with significantly lower EDP values after 75 min (end of sevo postC) and/or 100 min of perfusion, respectively, compared to hearts treated by I/R alone.

DP values after 100 min of perfusion, both in Controls (50.3 ± 5.0, p<0.01) and DHs (65.1 ± 2.9, p<0.05), were positively affected by addition of 3.6% sevo postC, and found to be significantly increased, compared to hearts treated by I/R alone (Ctr: 32.6 ± 3.2, Diab: 44.7 ± 4.5). The recovery of the DHs subjected to 3.6% sevo postC was found to be significantly faster and higher compared to Control (p<0.05). Additionally, DHs were found to have significantly (p<0.05) better recoveries for +dp/dt, -dp/dt and/or WI, after I/R and sevo postC compared to Control (see Table 2).

Both Control hearts and DHs subjected to 3.6% sevo postC, showed trends towards decreases in HR during anesthetic administration (peak at 75 min). This depressant effect of sevoflurane was further investigated in the healthy normoxic myocardium and revealed a rapid restoration of hemodynamic parameters after sevoflurane discontinuation (S1 Fig). These temporary depressant effects, may to some extent affect the hemodynamic changes observed during sevo administration. In Control hearts subjected to I/R + 3.6% sevo postC, HR recovery at the end of reperfusion was significantly higher compared to DH (99.3% ± 5.8 vs. 84.4 ± 3.7, P<0.05).

Administration of simvastatin to STZ-induced diabetic animals caused a worsening of recovery in all hemodynamic parameters in the DHs after sevo postC, and led to significant higher EDP values at the end of perfusion (70.0 ± 1.8, p<0.05), compared to DHs treated by I/R alone (48.1 ± 4.7; Table 2).

### Myocardial mRNA expression levels of eNOS, iNOS

As shown in Table 3, end of stabilization, eNOS mRNA expression levels were found to be significantly higher in DHs compared to Controls (in arbitrary units (AU), relative to Control baseline values). iNOS mRNA levels were also increased in DHs, but didn’t reach statistical significance.

**Table 3:** mRNAs expression levels of eNOS and iNOS in rat hearts at baseline (end of stabilization).

Parameter	Group	Perfusion
		Baseline (end of stabilization)
**eNOS**	**Control**	1.00 ± 0.06
	**Diabetic**	2.95 ± 0.41 *
**iNOS**	**Control**	1.00 ± 0.12
	**Diabetic**	1.75 ± 0.33

Data are presented as Mean (AU) ± SEM.

* denotes p<0.01 vs. Control.

As previously reported [21, 22], simvastatin (0.5 mg/kg; 2 day administration) significantly increased eNOS mRNA expression levels (2.19 ± 0.36, p<0.05), compared to vehicle treated rats (1.00 ± 0.21) [21]. iNOS mRNA expression levels also increased after simvastatin administration (1.81 ± 0.42), but did not reach statistical significance compared to vehicle treated rats (1.00 ± 0.10).

### Myocardial mRNA expression levels of H-ferritin and L-ferritin

The H- and L-ferritin mRNA ratio *within* Control and Diabetic hearts were investigated. L-ferritin mRNA levels (in AU, relative to H-ferritin baseline values) were found to be significantly lower compared to H-ferritin (the dominant ferritin-subunit in the heart) mRNA levels, at baseline (end of stabilisation), in both Control (0.35 ± 0.03 vs. 1.00 ± 0.18, p<0.05) and Diabetic hearts (0.49 ± 0.10 vs. 1.00 ±0.16, p<0.05).

Table 4 depicts H- and L-ferritin mRNA levels *between* Control and Diabetic hearts, along the various perfusion protocols. H-ferritin and L-ferritin mRNA expression levels (in AU, relative to Control baseline values; Table 4) were found to differ between Control and DHs at baseline. H-ferritin expression levels were lower (0.63 ± 0.06), whereas L-ferritin levels were found to be higher (1.34 ± 0.26) in DHs, compared to Controls (p = NS). They also differ in DHs during reperfusion. H-ferritin mRNA expression levels increased in DH along the perfusion protocols, and were higher (without reaching statistical significance) compared to Controls, particularly after I/R and I/R + 3.6% sevo postC. No differences in Control H-ferritin mRNA expression levels were detected between the different protocols after 120 min of perfusion.

**Table 4:** mRNAs levels of H- and L-subunits of ferritin in rat hearts subjected to the various perfusion protocols.

Parameter	Group	Protocol	Perfusion			
			Baseline (end of stabilization)	60 min (end of ischemia)	75 min (end of sevo postC)	120 min (end of reperfusion)
**H-Ft**	**Control**	**SHAM**	1.00 ± 0.18	0.50 ± 0.06 # ^	0.79 ± 0.18	0.94 ± 0.20
		**I/R**		1.00 ± 0.18	1.05 ± 0.13	1.02 ± 0.14
		**I/R + 3.6% sevo postC**			1.08 ± 0.19	0.92 ± 0.16
	**Diabetic**	**SHAM**	0.63 ± 0.06	0.51 ± 0.08	0.55 ± 0.09	1.25 ± 0.38
		**I/R**		0.70 ± 0.26	1.84 ± 0.60	1.80 ± 0.51
		**I/R + 3.6% sevo postC**			1.42 ± 0.34	1.60 ± 0.41
**L-Ft**	**Control**	**SHAM**	1.00 ± 0.08	0.39 ± 0.03 ^	0.70 ± 0.07	2.19 ± 0.61 *
		**I/R**		0.74 ± 0.15	0.58 ± 0.04	0.66 ± 0.10
		**I/R + 3.6% sevo postC**			0.59 ± 0.01 ^	0.61 ± 0.05 ^
	**Diabetic**	**SHAM**	1.34 ± 0.26	1.80 ± 0.30 ¥	2.37 ± 0.11 # ^ ¥	2.02 ± 0.17
		**I/R**		1.63 ± 0.20 ¥	1.42 ± 0.13 ¥	1.33 ± 0.29
		**I/R + 3.6% sevo postC**			1.50 ± 0.28 ¥	1.52 ± 0.34 ¥

Data are presented as Mean (AU) ± SEM.

* denotes p<0.01 vs. I/R in the respective group, # denotes p<0.05 vs. I/R in the respective group, ¥ denotes p<0.05 vs. the respective protocol in Control. ^ denotes p<0.05 vs. baseline in the respective group.

In Control SHAM perfused hearts, L-ferritin mRNA expression levels dropped significantly after 60 min of perfusion (0.39 ± 0.03, p<0.05), compared to baseline (1.00 ± 0.08). After 120 min of perfusion, expression levels increased significantly to 2.19 ±0.61 (p<0.01), compared to I/R (0.66 ± 0.10). In SHAM DHs, L-ferritin levels also increased during the course of the perfusion protocol, and were found to be significantly higher after 75 min of perfusion (2.37 ± 0.11, p<0.05) compared to baseline (1.34 ± 0.26) and I/R (1.42 ± 0.13). However, Control hearts treated by I/R alone and I/R + 3.6% sevo postC had reduced L-ferritin levels after 75 min and 120 min of perfusion, compared to baseline. Thus, L-ferritin mRNA expression levels (as H-ferritin mRNA levels) were mostly higher in DHs compared to Control hearts, and reached statistical significance depending on the time point and perfusion protocol. In addition to ferritin, heme-oxygenase 1 (HO-1) mRNA levels, another protein associated with both iron metabolism and oxidative stress, was also found to be upregulated in DHs, compared to Control hearts (S1 Table).

### Myocardial ferritin protein levels

**Fig 3:**
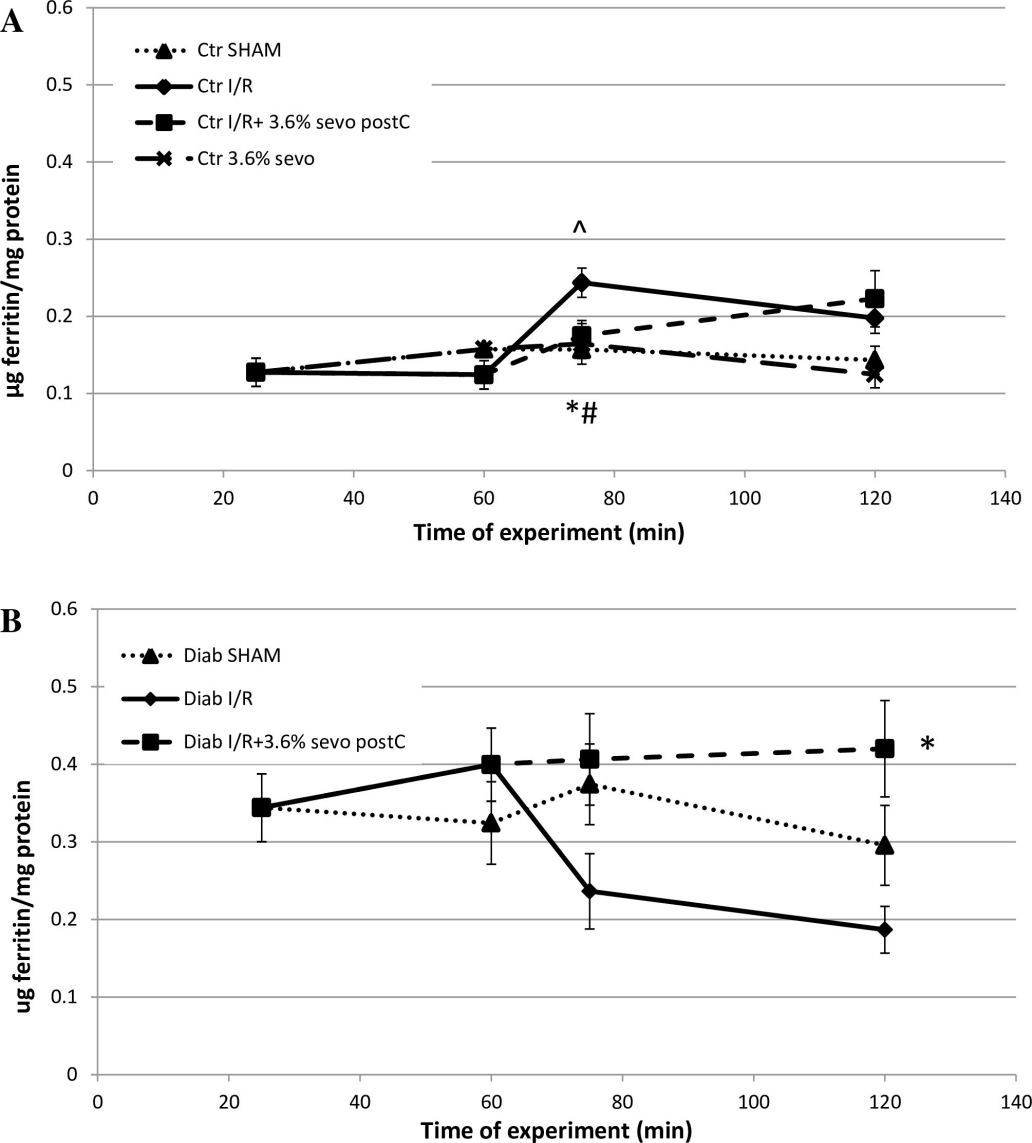
Myocardial ferritin protein levels in normoglycemic control hearts and in STZ-induced diabetic hearts. (A) Ferritin protein levels in Control hearts and STZ-induced DHs (B) along different time points of the perfusion protocols. Data are presented as Mean ± SEM and indicate the heart ferritin. * denotes p<0.01 vs. I/R. # denotes p<0.05 vs. I/R. ^ denotes p<0.05 vs. baseline.

Myocardial ferritin protein levels (in μg ferritin/mg protein) were measured along different time points of the various perfusion protocols and are depicted in Fig 3. In the Control hearts, baseline ferritin levels were 0.127 ± 0.018 (Fig 3A). Compared to baseline (end of stabilization), ferritin protein levels were significantly upregulated during the first 15 min of reperfusion (0.244 ± 0.019, p<0.05) in the hearts subjected to I/R alone. PostC with 3.6% sevoflurane, was administered during the first 15 min of reperfusion, and effectively prevented the increase (0.175 ± 0.020, p<0.05 vs. I/R) in heart ferritin protein levels. After discontinuation of the sevoflurane, ferritin levels in the I/R + 3.6% sevo group reached similar levels to those in Control hearts treated by I/R alone (0.222 ± 0.036 vs. 0.198 ± 0.020, respectively).

In the DHs, baseline ferritin protein levels were 2.7-fold higher compared to Control non-diabetic hearts (0.344 ± 0.044 vs. 0.127 ± 0.018; p<0.01; Fig 3B). In DHs subjected to I/R alone, ferritin protein levels decreased to 0.187 ± 0.030 after 120 min of perfusion. PostC with 3.6% sevofllurane prevented this decrease in ferritin protein levels (0.420 ± 0.062, p<0.01 vs I/R).

## Discussion

In the clinical situation, the protective benefits of PC have not yet been realized, not at least because PC can only be implemented in the situations where the ischemic events can be predicted, which in the case of an acute MI is unattainable. Pharmacological postC with volatile anestetics, such as sevoflurane, is an attractive and amenable approach in cardiac surgery, as it can be administered at the onset of reperfusion and does not require additional ischemic periods to a heart that is already at risk.

In the healthy rat heart we previously reported that the cardioprotection by IPC involves the generation of an iron signal, through activation of the proteasome, which results in the *de novo* synthesis and accumulation of ferritin [4, 27]. The important signalling molecule NO [33, 34], depending on its concentration, and its bio-active redox form, was found to regulate iron metabolism and play a role in myocardial protection against I/R-injury [28]. In the STZ-induced DH however, impaired iron homeostasis was found to lead to loss of the IPC generated myocardial protection [10]. The current study investigated whether alterations in iron homeostasis and ferritin accumulation are also involved in the cardioprotective effects generated by sevo postC, and whether these effects can be salvaged in the STZ-induced DH by simvastatin, through increasing NO bioavailability.

Infarct size analyses of isolated rat hearts revealed that sevo postC significantly reduced myocardial infarct size, in a dose-dependent matter in healthy normoglycemic Control hearts, but not in DHs (Fig 1). These results support previous findings and show that cardioprotective interventions, employed to reduce I/R-injury, are abrogated in the diabetic and hyperglycemic heart [5, 10, 14, 35]. Previously, we showed that this abrogation of sevo postC in STZ-induced diabetic animals, using an *in vivo* model of myocardial I/R, was associated with decreased STAT3 phophorylation/activation and involves the mK_ATP_ channel [5]. Even though the underlying mechanisms of postC (and PC) are highly complex and remain only partly understood, a decrease in STAT3 activation could be associated with decreased NO availability, as various signalling cascades (JAK/STAT, protein kinase C/MAPK/ERK) involved in STAT3 activation are NO regulated [8, 36]. Indeed, increasing NO bioavailability through simavastatin administration was previously found to reduce myocardial injury and restore the cardioprotective effects of IPC in diabetic db/db mice and hyperglycemic mongrel dogs, respectively [21, 22]. These cardioprotective effects were found to be independent of the cholesterol-lowering effect of simvastatin [21, 22]. Numerous other experimental and clinical studies have reported on the pleiotropic beneficial effects of statins in relation to myocardial cardioprotection and/or protection against I/R-injury [37]. Interestingly, the data presented here revealed that DHs of simvastatin pre-treated STZ-induced diabetic animals, respond differently to the conditioning stimulus and tend to have larger infarct sizes (i.e. I/R + 3.6% sevo postC; 35.2% ± 1.2), when compared to DHs treated by I/R alone (26.7% ± 2.2).

Functional myocardial recovery analyses revealed that both Control and DHs, treated with I/R + 3.6% sevo postC, show better recoveries for various hemodynamic parameters, compared to hearts treated by I/R alone (Table 2). These results suggest that even though sevo postC doesn't reduce infarct size in DHs, it can positively affect functional myocardial recovery. Many studies have reported on the depressant myocardial hemodynamic effects of volatile anesthetics (S1 Fig). This depressant effect might improve myocardial contraction after I/R-injury, however, it is thought not to be responsible for the observed sevo postC induced reduction of myocardial infarct size [11, 38–40]. In line with this, the depressant effects of sevoflurane are also detected in DH, where it was not associated with a reduction in infarct size. Note that, in the current study two sevoflurane concentrations (2.4% and 3.6%) were tested. These clinically relevant concentrations are commonly employed during *in vivo* and *ex vivo* isolated rat heart postconditioning studies [7, 41–43], and were used here to investigate whether simvastatin can rescue sevo postC in isolated diabetic rat hearts. Tanaka *et al*. showed that PC with increased isoflurane concentrations could overcome the depressant effect of hyperglycemia on infarct size reduction, in hyperglycemic dogs [11]. It was not further investigated herein, and can therefore not be ruled out, whether higher (>3.6%) sevoflurane concentrations alone can rescue cardioprotection in DH. It was previously reported, however, that 1 MAC (~2.5%) of sevoflurane induces maximal cardioprotection by postconditioning *in vivo* in rats. Higher sevoflurane concentrations only increased the observed cardiodepressant side effects [38]. Further analyses of the hemodynamic parameters revealed that STZ-induced DHs had significantly lower HR, +dp/dt, -dp/dt and WI baseline values compared to Control hearts. Functional recoveries of several of these parameters were better, along the various perfusion protocols, in DHs than Controls, even though infarct sizes tend to be larger [10, 44–46].

Previously, bradycardia and contractile hyporesponsiveness to ß-adrenergic stimulation, in STZ-induced diabetic rats, could be normalized in the presence of the non-selective NOS-inhibitor L-NAME. It was therefore suggested that the observed depressant effects (8 weeks after induction) were mediated by increased NO levels [45]. In line with these findings, Stockklauser-Färber *et al*. detected temporary increases in NOS activity and eNOS mRNA expression levels, from 4 weeks after induction of diabetes. Significantly lower NOS activity and eNOS mRNA levels were only observed after 46 weeks of diabetes. It was suggested that the temporary increases are caused by the enhanced oxidated stress exerted by the diabetic and hyperglycemic state [47]. Farhankhoee *et al*. also observed increased oxidative stress and increased iNOS and eNOS mRNA levels 4 weeks after induction of diabetes by STZ. Interestingly, this upregulation was not found to coincide with increased NO formation, and it was therefore suggested that the highly reactive NO is quickly scavenged by superoxide anions (O_2_^-^) to form the potentially damaging peroxynitrite (ONOO^-^) [48]. The current study also detected significant increases in eNOS mRNA expression levels in the rat myocardium, 4 weeks after the induction of diabetes by STZ (Table 3), that can explain the observed bradycardia in DHs (Table 2). Simvastatin pre-treatment, likely further increased NO and/or ONOO^-^ levels, and thus caused aggravation rather than reduction of myocardial injury, after sevo postC in DHs of STZ-induced diabetic rats. As simvastatin pre-treatment was not found to rescue cardioprotection by sevo postC, no further animals were pre-treated with simvastatin. Furthermore, in the current study NOS levels were analysed to confirm the NO-inducing effects of simvastatin, elucidate the observed baseline bradycardia in STZ-induced DHs, and explain the observed aggravation of myocardial injury after simvastatin pre-treatment. Additional NO bioavailability/activity analyses were beyond the scope of this study. Exogenous NO was previously found to play a role in myocardial protection and ferritin protein accumulation in the healthy rat heart [28]. Future studies will thus need to elucidate whether simvastatin treatment alone can evoke similar ferritin mediated protective effects, through induction of NOS, in healthy or DHs. Interestingly, simvastatin was previously shown to ameliorate diabetic cardiomyopathy and/or myocardial dysfunction in STZ-induced diabetic animals [49, 50], indicating it can mediate cardioprotection in this animal model, despite the high NO-levels. In the current study animals received simvastatin for 2 consecutive days before the start of the experiments, as this was found to already significantly increase eNOS mRNA levels. Although not further investigated here, it is possible that extended or acute simvastatin dosages differently affect the response of the STZ-induced DH to I/R + sevo postC and so could mediate a cardioprotective effect [37, 49, 50]. Nevertheless, other diabetic animal models (e.g. db/db mice) may be employed to further investigate salvaging of myocardial protection by simvastatin pre-treatment together with a postconditioning stimulus, such as sevo postC. In addition to increased iNOS and eNOS mRNA levels in STZ-induced rat hearts, Farhangkhoee *et al*. also found that diabetes induced oxidative stress was associated with upregulated HO-1 [48]. Contradictory roles for HO-1 have been reported in the cardiovascular complications of diabetes, with some studies reporting pro-oxidant activities and others reporting anti-oxidant activities of HO-1 [46, 48, 51]. Although the HO enzyme system wasn’t fully analysed here, in line with Farhangkhoee *et al*., HO-1 mRNA expression levels were found to be upregulated in hearts of 4 week STZ-induced diabetic animals, compared to Control animals (S1 Table). Furthermore, SHAM hearts were found to have higher HO-1 mRNA levels compared to hearts subjected to I/R alone and/or I/R + 3.6% sevo postC, suggesting a dual role of HO-1, that could involve redox-active iron [48].

Iron, an essential consistent of many macromolecules, is involved in energy production, respiration and metabolism. Excess redox-active iron however, can enhance ROS-induced oxidative damage through the formation of hydroxyl radicals and shift the redox balance from cell survival to cell death [23, 24]. Because of its redox activity, most of the cellular iron is protein bound by ferritin. Elevated levels of ferritin have so been found to provide functional protection against oxidative stress, including I/R-injury [4, 32]. Syntheses of ferritin subunits (i.e. H- and L-ferritin) are regulated transcriptionally and post-transcriptionally. Increases in gene transcription of the ferritin mRNA subunits are observed in the presence of excessive iron molecules and oxidative stress [52, 53]. Ferritin protein levels are post-transcriptionally regulated by the iron regulatory proteins (IRPs) 1 and 2. When iron is abundant, IRP1 and 2 dissociate from the ferritin mRNA, allowing its synthesis [54]. In the current study, minor changes were observed in H- and L-ferrtin mRNA levels along the various perfusion protocols in both Control and DHs (Table 4). These findings are in contrast to our earlier observations, that showed a marked ~4-6-fold increase in L-ferritin mRNA levels, directly after IPC + ischemia in healthy Control hearts, and a ~2-fold decrease of H-ferritin mRNA (generally the more dominant subunit in the heart) after IPC in DHs [4, 10]. The mRNA data presented here suggest that, unlike IPC, sevo postC doesn't provoke a transient iron and/or ROS signal that causes ferritin mRNA synthesis [52, 53]. Future studies measuring markers of oxidative stress are required to confirm this hypothesis. Albeit not evident by the mRNA levels, the resting diabetic myocardium was found to contain double the amount of ferritin protein than the healthy Control heart (Fig 3). These findings are in good agreement with previous observations of the diabetic state, and are likely to be a response to prolonged increased levels of redox-active free iron and oxidative stress [10, 48]. The current study also revealed that the cardioprotective effect of sevo postC is not associated with an increase in *de novo* ferritin protein synthesis (Fig 3A) [4, 5, 7]. Control hearts showed a 2-fold significant transient increase in ferritin protein levels after ischemia + early (15 min.) reperfusion, but not after ischemia + sevo postC. Although this increase in ferritin protein levels is not as prominent as our previously observed ~5-6-fold increase after IPC + ischemia [4, 10], it does indicate transient elevated free-iron levels and subsequent ferritin translation [54]. Contrarily to Control hearts, DHs subjected to ischemia + early (15 min.) reperfusion revealed a two-fold decrease in ferritin levels (Fig 3A and 3B). Sevo postC was found to prevent this ferritin degradation (Fig 3B), even though it did not salvage myocardial protection against I/R-injury, in the STZ-induced DHs. Earlier, we showed that in healthy animals the IPC-mediated iron-signal and subsequent ferritin accumulation, involved prior proteasome-mediated ferritin degradation [27]. Enhanced protein degradation, including ferritin, has also been reported in diabetes and particularly ischemia [10, 55–57]. Inhibition of the ferritin degradation by protease inhibitors, was shown to rescue IPC mediated myocardial protection in STZ-induced DHs [10]. Whether sevo postC plays a role in the preventing proteasome mediated protein degradation, possibly by decreasing oxidative stress levels, remains to be elucidated. Note, although not measured here, it is possible that the available non-degraded ferritin protein reached saturation levels, and was unable to chelate the excess intracellular redox-active free iron present in the DH [10, 28, 48].

In conclusion, simvastatin did not salvage sevo postC in isolated STZ-induced DHs. In healthy hearts, unlike IPC, sevo postC mediated cardioprotection but does not involve *de novo* ferritin synthesis and accumulation [4]. Despite the known mechanistic overlap between cardioprotective interventions, like IPC and sevo postC, distinct mechanistic differences occur and remain to be elucidated [8, 58]. Further studies are also required in order to elucidate whether ferritin mediated cardioprotection is IPC specific, or can also be induced by e.g. simvastatin treatment, PC with volatile anesthetics, such as sevoflurane, and/or various postC stimuli.

## Supporting information

S1 Fig The effects of 3.6% sevoflurane on the hemodynamics of normoglycemic control hearts. (A) Heart rate (HR) and (B) Developed Pressure (DP) of the normoxic Control hearts during continues perfusion with or without 3.6% sevoflurane (sevo). Sevo was administered from 60–75 min of perfusion. Data are presented as percent changes from baseline (Mean ± SEM). * denotes p<0.01 vs. SHAM.
(PDF)

S1 Table mRNAs expression levels of HO-1 in rat hearts subjected to various perfusion protocols. Data are presented as Mean (AU) ± SEM. # denotes p<0.05 vs. SHAM in the respective group, ^ denotes p<0.05 vs. baseline in the same group, ¥ denotes p<0.05 vs. the respective protocol in Control. HO-1 (NM_012580.2) real-time PCR was conducted, as described in 'Materials and Methods', using the following primers; Fw-primer: ACAGAGTTTCTTCGCCAGAGG, Rv-primer: GGGGGCCAACACTGCATTTA, and normalized to β-actin.
(PDF)

## References

[1] Murry CE, Jennings RB, Reimer KA. Preconditioning with ischemia: a delay of lethal cell injury in ischemic myocardium. Circulation. 1986;74(5):1124–36. Epub 1986/11/01. . 3769170 10.1161/01.cir.74.5.1124

[2] Zhao ZQ, Corvera JS, Halkos ME, Kerendi F, Wang NP, Guyton RA, et al Inhibition of myocardial injury by ischemic postconditioning during reperfusion: comparison with ischemic preconditioning. Am J Physiol Heart Circ Physiol. 2003;285(2):H579–88. Epub 2003/07/16. 10.1152/ajpheart.01064.2002 [pii]. . 12860564

[3] Brandenburger T, Grievink H, Heinen N, Barthel F, Huhn R, Stachuletz F, et al Effects of remote ischemic preconditioning and myocardial ischemia on microRNA-1 expression in the rat heart in vivo. Shock. 2014;42(3):234–8. Epub 2014/07/01. 10.1097/SHK.0000000000000201 . 24978894

[4] Chevion M, Leibowitz S, Aye NN, Novogrodsky O, Singer A, Avizemer O, et al Heart protection by ischemic preconditioning: a novel pathway initiated by iron and mediated by ferritin. J Mol Cell Cardiol. 2008;45(6):839–45. Epub 2008/09/27. S0022-2828(08)00580-4 [pii] 10.1016/j.yjmcc.2008.08.011 . 18817783

[5] Drenger B, Ostrovsky IA, Barak M, Nechemia-Arbely Y, Ziv E, Axelrod JH. Diabetes blockade of sevoflurane postconditioning is not restored by insulin in the rat heart: phosphorylated signal transducer and activator of transcription 3- and phosphatidylinositol 3-kinase-mediated inhibition. Anesthesiology. 2011;114(6):1364–72. Epub 2011/03/04. 10.1097/ALN.0b013e31820efafd . 21368653

[6] Warltier DC, al-Wathiqui MH, Kampine JP, Schmeling WT. Recovery of contractile function of stunned myocardium in chronically instrumented dogs is enhanced by halothane or isoflurane. Anesthesiology. 1988;69(4):552–65. Epub 1988/10/01. . 3177915 10.1097/00000542-198810000-00016

[7] Gong JS, Yao YT, Fang NX, Li LH. Sevoflurane postconditioning attenuates reperfusion-induced ventricular arrhythmias in isolated rat hearts exposed to ischemia/reperfusion injury. Mol Biol Rep. 2012;39(6):6417–25. Epub 2012/03/27. 10.1007/s11033-012-1447-9 . 22447537

[8] Hausenloy DJ, Yellon DM. Preconditioning and postconditioning: underlying mechanisms and clinical application. Atherosclerosis. 2009;204(2):334–41. Epub 2008/12/17. S0021-9150(08)00748-X [pii] 10.1016/j.atherosclerosis.2008.10.029 . 19081095

[9] Ge ZD, Li Y, Qiao S, Bai X, Warltier DC, Kersten JR, et al Failure of Isoflurane Cardiac Preconditioning in Obese Type 2 Diabetic Mice Involves Aberrant Regulation of MicroRNA-21, Endothelial Nitric-oxide Synthase, and Mitochondrial Complex I. Anesthesiology. 2017 Epub 2017/10/19. 10.1097/ALN.0000000000001926 . 29040168 PMC5726897

[10] Vinokur V, Berenshtein E, Bulvik B, Grinberg L, Eliashar R, Chevion M. The bitter fate of the sweet heart: impairment of iron homeostasis in diabetic heart leads to failure in myocardial protection by preconditioning. PLoS One. 2013;8(5):e62948 Epub 2013/05/22. 10.1371/journal.pone.0062948 PONE-D-13-06379 [pii]. . 23690966 PMC3655153

[11] Tanaka K, Kehl F, Gu W, Krolikowski JG, Pagel PS, Warltier DC, et al Isoflurane-induced preconditioning is attenuated by diabetes. Am J Physiol Heart Circ Physiol. 2002;282(6):H2018–23. Epub 2002/05/11. 10.1152/ajpheart.01130.2001 . 12003806

[12] Povlsen JA, Lofgren B, Rasmussen LE, Nielsen JM, Norregaard R, Kristiansen SB, et al Cardioprotective effect of L-glutamate in obese type 2 diabetic Zucker fatty rats. Clin Exp Pharmacol Physiol. 2009;36(9):892–8. Epub 2009/03/21. CEP5166 [pii] 10.1111/j.1440-1681.2009.05166.x . 19298538

[13] Tsang A, Hausenloy DJ, Mocanu MM, Carr RD, Yellon DM. Preconditioning the diabetic heart: the importance of Akt phosphorylation. Diabetes. 2005;54(8):2360–4. . 16046302 10.2337/diabetes.54.8.2360

[14] Whittington HJ, Babu GG, Mocanu MM, Yellon DM, Hausenloy DJ. The diabetic heart: too sweet for its own good? Cardiol Res Pract. 2012:845698 Epub 2012/03/31. 10.1155/2012/845698 . 22462028 PMC3296224

[15] Kersten JR, Montgomery MW, Ghassemi T, Gross ER, Toller WG, Pagel PS, et al Diabetes and hyperglycemia impair activation of mitochondrial K(ATP) channels. Am J Physiol Heart Circ Physiol. 2001;280(4):1744–50. 10.1152/ajpheart.2001.280.4.H1744 . 11247788

[16] Sivaraman V, Hausenloy DJ, Wynne AM, Yellon DM. Preconditioning the diabetic human myocardium. J Cell Mol Med. 2010;14(6B):1740–6. 10.1111/j.1582-4934.2009.00796.x . 19508386 PMC3829035

[17] Hotta H, Miura T, Miki T, Togashi N, Maeda T, Kim SJ, et al Angiotensin II type 1 receptor-mediated upregulation of calcineurin activity underlies impairment of cardioprotective signaling in diabetic hearts. Circ Res. 2010;106(1):129–32. 10.1161/CIRCRESAHA.109.205385 . 19910577

[18] Cai S, Khoo J, Mussa S, Alp NJ, Channon KM. Endothelial nitric oxide synthase dysfunction in diabetic mice: importance of tetrahydrobiopterin in eNOS dimerisation. Diabetologia. 2005;48(9):1933–40. Epub 2005/07/22. 10.1007/s00125-005-1857-5 . 16034613

[19] Komers R, Schutzer WE, Reed JF, Lindsley JN, Oyama TT, Buck DC, et al Altered endothelial nitric oxide synthase targeting and conformation and caveolin-1 expression in the diabetic kidney. Diabetes. 2006;55(6):1651–9. Epub 2006/05/30. 55/6/1651 [pii] 10.2337/db05-1595 . 16731827

[20] Okon EB, Chung AW, Rauniyar P, Padilla E, Tejerina T, McManus BM, et al Compromised arterial function in human type 2 diabetic patients. Diabetes. 2005;54(8):2415–23. Epub 2005/07/28. 54/8/2415 [pii]. . 16046309 10.2337/diabetes.54.8.2415

[21] Lefer DJ, Scalia R, Jones SP, Sharp BR, Hoffmeyer MR, Farvid AR, et al HMG-CoA reductase inhibition protects the diabetic myocardium from ischemia-reperfusion injury. FASEB J. 2001;15(8):1454–6. Epub 2001/06/02. . 11387255 10.1096/fj.00-0819fje

[22] Gu W, Kehl F, Krolikowski JG, Pagel PS, Warltier DC, Kersten JR. Simvastatin restores ischemic preconditioning in the presence of hyperglycemia through a nitric oxide-mediated mechanism. Anesthesiology. 2008;108(4):634–42. Epub 2008/03/26. 10.1097/ALN.0b013e3181672590 00000542-200804000-00015 [pii]. . 18362595 PMC4378683

[23] Breuer W, Shvartsman M, Cabantchik ZI. Intracellular labile iron. Int J Biochem Cell Biol. 2008;40(3):350–4. Epub 2007/04/25. S1357-2725(07)00090-8 [pii] 10.1016/j.biocel.2007.03.010 . 17451993

[24] Chevion M. A site-specific mechanism for free radical induced biological damage: the essential role of redox-active transition metals. Free Radic Biol Med. 1988;5(1):27–37. Epub 1988/01/01. . 3075945 10.1016/0891-5849(88)90059-7

[25] Berenshtein E, Mayer B, Goldberg C, Kitrossky N, Chevion M. Patterns of mobilization of copper and iron following myocardial ischemia: possible predictive criteria for tissue injury. J Mol Cell Cardiol. 1997;29(11):3025–34. Epub 1998/02/28. S0022-2828(97)90535-6 [pii] 10.1006/jmcc.1997.0535 . 9405177

[26] Chevion M, Jiang Y, Har-El R, Berenshtein E, Uretzky G, Kitrossky N. Copper and iron are mobilized following myocardial ischemia: possible predictive criteria for tissue injury. Proc Natl Acad Sci U S A. 1993;90(3):1102–6. Epub 1993/02/01. . 8430081 10.1073/pnas.90.3.1102 PMC45819

[27] Bulvik BE, Berenshtein E, Meyron-Holtz EG, Konijn AM, Chevion M. Cardiac protection by preconditioning is generated via an iron-signal created by proteasomal degradation of iron proteins. PLoS One. 2012;7(11):e48947 Epub 2012/11/17. 10.1371/journal.pone.0048947 PONE-D-12-19817 [pii]. . 23155431 PMC3498359

[28] Grievink H, Zeltcer G, Drenger B, Berenshtein E, Chevion M. Protection by Nitric Oxide Donors of Isolated Rat Hearts Is Associated with Activation of Redox Metabolism and Ferritin Accumulation. PLoS One. 2016;11(7):e0159951 Epub 2016/07/23. 10.1371/journal.pone.0159951 PONE-D-16-13952 [pii]. . 27447933 PMC4957751

[29] Gorbe A, Varga ZV, Kupai K, Bencsik P, Kocsis GF, Csont T, et al Cholesterol diet leads to attenuation of ischemic preconditioning-induced cardiac protection: the role of connexin 43. Am J Physiol Heart Circ Physiol. 2011;300(5):H1907–13. Epub 2011/03/15. ajpheart.01242.2010 [pii] 10.1152/ajpheart.01242.2010 . 21398600

[30] Song T, Lv LY, Xu J, Tian ZY, Cui WY, Wang QS, et al Diet-induced obesity suppresses sevoflurane preconditioning against myocardial ischemia-reperfusion injury: role of AMP-activated protein kinase pathway. Exp Biol Med (Maywood). 2011;236(12):1427–36. Epub 2011/11/15. ebm.2011.011165 [pii] 10.1258/ebm.2011.011165 . 22075552

[31] Livak KJ, Schmittgen TD. Analysis of relative gene expression data using real-time quantitative PCR and the 2(-Delta Delta C(T)) Method. Methods. 2001;25(4):402–8. Epub 2002/02/16. 10.1006/meth.2001.1262 [pii]. . 11846609

[32] Berenshtein E, Vaisman B, Goldberg-Langerman C, Kitrossky N, Konijn AM, Chevion M. Roles of ferritin and iron in ischemic preconditioning of the heart. Mol Cell Biochem. 2002;234-235(1–2):283–92. Epub 2002/08/07. . 12162445

[33] Cary SP, Winger JA, Derbyshire ER, Marletta MA. Nitric oxide signaling: no longer simply on or off. Trends Biochem Sci. 2006;31(4):231–9. 10.1016/j.tibs.2006.02.003 . 16530415

[34] Mustafa AK, Gadalla MM, Snyder SH. Signaling by gasotransmitters. Sci Signal. 2009;2(68):re2 10.1126/scisignal.268re2 . 19401594 PMC2744355

[35] Huhn R, Heinen A, Weber NC, Hollmann MW, Schlack W, Preckel B. Hyperglycaemia blocks sevoflurane-induced postconditioning in the rat heart in vivo: cardioprotection can be restored by blocking the mitochondrial permeability transition pore. Br J Anaesth. 2008;100(4):465–71. Epub 2008/02/29. aen022 [pii] 10.1093/bja/aen022 . 18305078

[36] Bolli R, Dawn B, Xuan YT. Role of the JAK-STAT pathway in protection against myocardial ischemia/reperfusion injury. Trends Cardiovasc Med. 2003;13(2):72–9. Epub 2003/02/15. S105017380200230X [pii]. . 12586443 10.1016/s1050-1738(02)00230-x

[37] Ludman A, Venugopal V, Yellon DM, Hausenloy DJ. Statins and cardioprotection—more than just lipid lowering? Pharmacology & therapeutics. 2009;122(1):30–43. Epub 2009/03/26. S0163-7258(09)00004-7 [pii] 10.1016/j.pharmthera.2009.01.002 . 19318042

[38] Obal D, Preckel B, Scharbatke H, Mullenheim J, Hoterkes F, Thamer V, et al One MAC of sevoflurane provides protection against reperfusion injury in the rat heart in vivo. Br J Anaesth. 2001;87(6):905–11. Epub 2002/03/07. . 11878695 10.1093/bja/87.6.905

[39] Park WK, Pancrazio JJ, Suh CK, Lynch C, 3rd. Myocardial depressant effects of sevoflurane. Mechanical and electrophysiologic actions in vitro. Anesthesiology. 1996;84(5):1166–76. Epub 1996/05/01. . 8624011 10.1097/00000542-199605000-00019

[40] Schlack W, Preckel B, Barthel H, Obal D, Thamer V. Halothane reduces reperfusion injury after regional ischaemia in the rabbit heart in vivo. Br J Anaesth. 1997;79(1):88–96. Epub 1997/07/01. S0007-0912(17)39910-5 [pii]. . 9301395 10.1093/bja/79.1.88

[41] Schlack W, Preckel B, Stunneck D, Thamer V. Effects of halothane, enflurane, isoflurane, sevoflurane and desflurane on myocardial reperfusion injury in the isolated rat heart. Br J Anaesth. 1998;81(6):913–9. . 10211019 10.1093/bja/81.6.913

[42] Yao Y, Li L, Gao C, Shi C. Sevoflurane postconditioning protects chronically-infarcted rat hearts against ischemia-reperfusion injury by activation of pro-survival kinases and inhibition of mitochondrial permeability transition pore opening upon reperfusion. Biol Pharm Bull. 2009;32(11):1854–61. Epub 2009/11/03. JST.JSTAGE/bpb/32.1854 [pii]. . 19881297 10.1248/bpb.32.1854

[43] Yao YY, Zhu MH, Zhang FJ, Wen CY, Ma LL, Wang WN, et al Activation of Akt and cardioprotection against reperfusion injury are maximal with only five minutes of sevoflurane postconditioning in isolated rat hearts. J Zhejiang Univ Sci B. 2013;14(6):511–7. Epub 2013/06/05. 10.1631/jzus.B1200195 . 23733428 PMC3682167

[44] Malone MA, Schocken DD, Hanna SK, Liang X, Malone JI. Diabetes-induced bradycardia is an intrinsic metabolic defect reversed by carnitine. Metabolism. 2007;56(8):1118–23. Epub 2007/07/10. S0026-0495(07)00140-0 [pii] 10.1016/j.metabol.2007.04.005 . 17618959

[45] Smith JM, Paulson DJ, Romano FD. Inhibition of nitric oxide synthase by L-NAME improves ventricular performance in streptozotocin-diabetic rats. J Mol Cell Cardiol. 1997;29(9):2393–402. Epub 1997/09/23. S0022-2828(97)90474-0 [pii] 10.1006/jmcc.1997.0474 . 9299363

[46] Cao J, Vecoli C, Neglia D, Tavazzi B, Lazzarino G, Novelli M, et al Cobalt-Protoporphyrin Improves Heart Function by Blunting Oxidative Stress and Restoring NO Synthase Equilibrium in an Animal Model of Experimental Diabetes. Front Physiol. 2012;3:160 Epub 2012/06/08. 10.3389/fphys.2012.00160 . 22675305 PMC3366474

[47] Stockklauser-Farber K, Ballhausen T, Laufer A, Rosen P. Influence of diabetes on cardiac nitric oxide synthase expression and activity. Biochim Biophys Acta. 2000;1535(1):10–20. Epub 2000/12/13. S0925-4439(00)00078-8 [pii]. . 11113627 10.1016/s0925-4439(00)00078-8

[48] Farhangkhoee H, Khan ZA, Mukherjee S, Cukiernik M, Barbin YP, Karmazyn M, et al Heme oxygenase in diabetes-induced oxidative stress in the heart. J Mol Cell Cardiol. 2003;35(12):1439–48. Epub 2003/12/05. S0022282803002888 [pii]. . 14654370 10.1016/j.yjmcc.2003.09.007

[49] Al-Rasheed NM, Al-Rasheed NM, Hasan IH, Al-Amin MA, Al-Ajmi HN, Mohamad RA, et al Simvastatin Ameliorates Diabetic Cardiomyopathy by Attenuating Oxidative Stress and Inflammation in Rats. Oxidative medicine and cellular longevity. 2017;2017:1092015 10.1155/2017/1092015 29138670 PMC5613468

[50] Thirunavukkarasu M, Selvaraju V, Dunna NR, Foye JL, Joshi M, Otani H, et al Simvastatin treatment inhibits hypoxia inducible factor 1-alpha-(HIF-1alpha)-prolyl-4-hydroxylase 3 (PHD-3) and increases angiogenesis after myocardial infarction in streptozotocin-induced diabetic rat. International journal of cardiology. 2013;168(3):2474–80. 10.1016/j.ijcard.2013.03.005 . 23590933

[51] Zhao Y, Zhang L, Qiao Y, Zhou X, Wu G, Wang L, et al Heme oxygenase-1 prevents cardiac dysfunction in streptozotocin-diabetic mice by reducing inflammation, oxidative stress, apoptosis and enhancing autophagy. PLoS One. 2013;8(9):e75927 Epub 2013/10/03. 10.1371/journal.pone.0075927 PONE-D-12-40642 [pii]. . 24086665 PMC3782439

[52] Cairo G, Tacchini L, Pogliaghi G, Anzon E, Tomasi A, Bernelli-Zazzera A. Induction of ferritin synthesis by oxidative stress. Transcriptional and post-transcriptional regulation by expansion of the "free" iron pool. J Biol Chem. 1995;270(2):700–3. Epub 1995/01/13. . 7822298 10.1074/jbc.270.2.700

[53] Hintze KJ, Theil EC. DNA and mRNA elements with complementary responses to hemin, antioxidant inducers, and iron control ferritin-L expression. Proc Natl Acad Sci U S A. 2005;102(42):15048–52. Epub 2005/10/12. 0505148102 [pii] 10.1073/pnas.0505148102 . 16217041 PMC1257710

[54] Meyron-Holtz EG, Ghosh MC, Iwai K, LaVaute T, Brazzolotto X, Berger UV, et al Genetic ablations of iron regulatory proteins 1 and 2 reveal why iron regulatory protein 2 dominates iron homeostasis. EMBO J. 2004;23(2):386–95. Epub 2004/01/17. 10.1038/sj.emboj.7600041 [pii]. . 14726953 PMC1271751

[55] Lenk SE, Bhat D, Blakeney W, Dunn WA Jr. Effects of streptozotocin-induced diabetes on rough endoplasmic reticulum and lysosomes of rat liver. Am J Physiol. 1992;263(5 Pt 1):E856–62. Epub 1992/11/11. 10.1152/ajpendo.1992.263.5.E856 . 1443117

[56] Marfella R, Cacciapuoti F, Grassia A, Manfredi E, De Maio G, Caruso G, et al Role of the ubiquitin-proteasome system in carotid plaque instability in diabetic patients. Acta Cardiol. 2006;61(6):630–6. Epub 2007/01/09. 10.2143/AC.61.6.2017962 . 17205920

[57] Marfella R, Di Filippo C, Portoghese M, Siniscalchi M, Martis S, Ferraraccio F, et al The ubiquitin-proteasome system contributes to the inflammatory injury in ischemic diabetic myocardium: the role of glycemic control. Cardiovasc Pathol. 2009;18(6):332–45. Epub 2009/01/16. S1054-8807(08)00129-4 [pii] 10.1016/j.carpath.2008.09.008 . 19144543

[58] Van Allen NR, Krafft PR, Leitzke AS, Applegate RL 2nd, Tang J, Zhang JH. The role of Volatile Anesthetics in Cardioprotection: a systematic review. Med Gas Res. 2012;2(1):22 Epub 2012/08/30. 2045-9912-2-22 [pii] 10.1186/2045-9912-2-22 . 22929111 PMC3598931

