# Supplementary material for: Sevoflurane postconditioning is not mediated by ferritin accumulation and cannot be rescued by simvastatin in isolated streptozotocin-induced diabetic rat hearts
Source: PLoS One. 2019 Jan 25;14(1):e0211238. doi: 10.1371/journal.pone.0211238 (PMC6347357; doi:10.1371/journal.pone.0211238)

S1 Figure. The effects of 3.6% sevoflurane on the hemodynamics of normoglycemic Control hearts.

(A) Heart rate (HR) and (B) Developed Pressure (DP) of the normoxic Control hearts during continues perfusion with or without 3.6% sevoflurane (sevo). Sevo was administered from 60-75 min of perfusion. Data are presented as percent changes from baseline (Mean ± SEM). * denotes p<0.01 vs. SHAM.

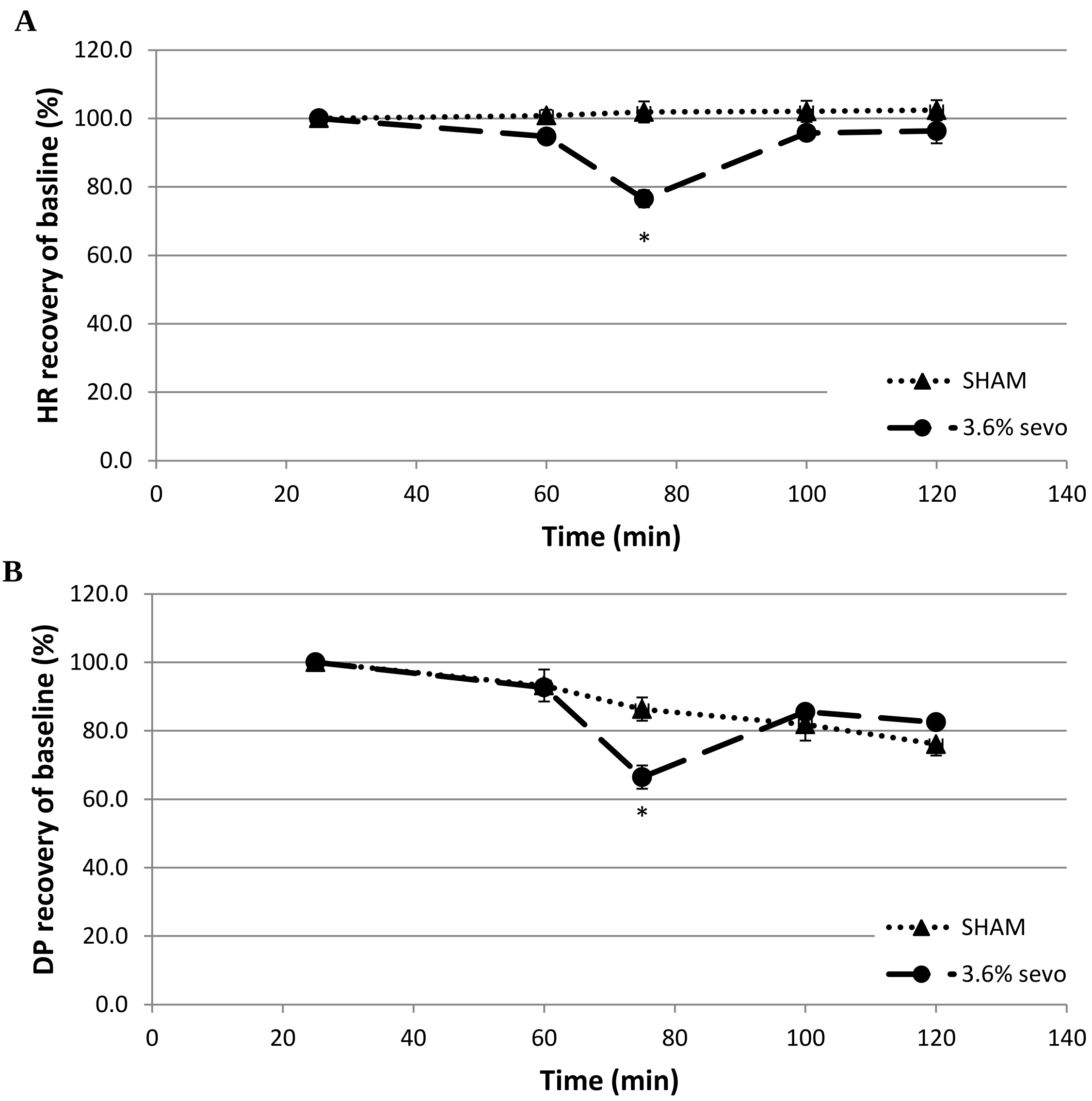

Supplement: S1 Fig — (A) Heart rate (HR) and (B) Developed Pressure (DP) of the normoxic Control hearts during continues perfusion with or without 3.6% sevoflurane (sevo). Sevo was administered from 60–75 min of perfusion. Data are presented as percent changes from baseline (Mean ± SEM). * denotes p<0.01 vs. SHAM. (PDF) [file pone.0211238.s001.pdf]
