# Supplementary material for: Sevoflurane postconditioning is not mediated by ferritin accumulation and cannot be rescued by simvastatin in isolated streptozotocin-induced diabetic rat hearts
Source: PLoS One. 2019 Jan 25;14(1):e0211238. doi: 10.1371/journal.pone.0211238 (PMC6347357; doi:10.1371/journal.pone.0211238)
Supplement: S1 Table — Data are presented as Mean (AU) ± SEM. # denotes p<0.05 vs. SHAM in the respective group, ^ denotes p<0.05 vs. baseline in the same group, ¥ denotes p<0.05 vs. the respective protocol in Control. HO-1 (NM_012580.2) real-time PCR was conducted, as described in 'Materials and Methods', using the following primers; Fw-primer: ACAGAGTTTCTTCGCCAGAGG, Rv-primer: GGGGGCCAACACTGCATTTA, and normalized to β-actin. (PDF) [file pone.0211238.s002.pdf]

S1 Table. mRNAs expression levels of HO-1 in rat hearts subjected to various perfusion protocols.

Data are presented as Mean (AU) ± SEM. # denotes $p<0.05$ vs. SHAM in the respective group, ^ denotes $p<0.05$ vs. baseline in the same group, ¥ denotes $p<0.05$ vs. the respective protocol in Control. HO-1 (NM_012580.2) real-time PCR was conducted, as described in 'Materials and Methods', using the following primers; Fw-primer: ACAGAGTTTCTTCGCCAGAGG, Rv-primer: GGGGGCCAACACTGCATTTA, and normalized to β-actin.

Group	Protocol	Perfusion		
		**Baseline (end of stabilization)**	**75 min (end of sevo postC)**	**120 min (end of reperfusion)**
**Control**	**SHAM**	1.00 ± 0.06	3.21 ± 0.47 ^	4.62 ± 0.66 ^
	**3.6% sevo**		1.90 ± 0.20 #	4.08 ± 1.03 ^
	**I/R**		1.70 ± 0.31 #	3.35 ± 0.19 ^
	**I/R + 3.6% sevo postC**		1.98 ± 0.23 #	3.80 ± 0.68 ^
**Diabetic**	**SHAM**	2.63 ± 0.69	5.53 ± 0.95	34.10 ± 9.36 ^ ¥
	**I/R + 3.6% sevo postC**		4.38 ± 1.74 ¥	9.90 ± 2.57 ¥

.
